# The impact of MET, IGF-1, IGF1R expression and *EGFR* mutations on survival of patients with non-small-cell lung cancer

**DOI:** 10.1371/journal.pone.0181527

**Published:** 2017-07-25

**Authors:** Samer Al-Saad, Elin Richardsen, Thomas K. Kilvaer, Tom Donnem, Sigve Andersen, Mehrdad Khanehkenari, Roy M. Bremnes, Lill-Tove Busund

**Affiliations:** 1 Institute of Medical Biology, UiT The Arctic University of Norway, Tromso, Norway; 2 Department of Clinical Pathology, University Hospital of Northern Norway, Tromso, Norway; 3 Institute of Clinical Medicine, UiT The Arctic University of Norway, Tromso, Norway; 4 Department of Oncology, University Hospital of Northern Norway, Tromso, Norway; University of South Alabama Mitchell Cancer Institute, UNITED STATES

## Abstract

**Introduction:**

To compare the efficacy of silver *in situ* hybridization (SISH) and immunohistochemistry (IHC) in detecting MET and IGF1R alterations and to investigate their prevalence and prognostic significance. A possible correlation between MET receptor expression, *MET* gene alterations and the two most frequent occurring *EGFR* gene mutations was also investigated.

**Materials and methods:**

Stage I to IIIA tumors from 326 patients with NSCLC were immunohistochemically tested for protein expression of MET and IGF-1. Their cytoplasmic expression was compared with the gene copy number of the *MET* and *IGF1R*genes by SISH in paraffin-embedded, formalin-fixed material. Correlations were made with the immunohistochemical expression of two frequent *EGFR* mutations and clinicopathological variables. Univariate and multivariate survival analyses was used to evaluate the prognostic efficacy of the tested markers.

**Results:**

In univariate analyses, high cytoplasmic MET expression showed a significant negative prognostic effect in adenocarcinoma patients (p = 0.026). *MET* gene to chromosome 7 ratio was a significant positive prognostic marker (p = 0.005), probably only due to the highly negative prognostic significance of chromosome 7 polysomy (p = 0.002). High *IGF1R* gene copy number was a negative prognostic marker for all NSCLC patients (p = 0.037). In the multivariate analysis, polysomy of chromosome 7 in tumor cells correlated significantly and independently with a poor prognosis (p = 0.011). In patients with adenocarcinoma, a high cytoplasmic MET expression was an independent negative prognostic marker (p = 0.013). In males a high *IGF1R* gene copy number to chromosome 15 count ratio was significantly and independently correlated to a poor prognosis (p = 0.018).

**Conclusion:**

MET protein expression provides superior prognostic information compared with SISH. Polysomy of chromosome 7 is an independent negative prognostic factor in NSCLC patients. This finding has an important implication while examining genes located on chromosome 7 by means of SISH. High *IGF1R* gene copy number to chromosome 15 count ratio is an independent predictor of inferior survival in male patients with primary NSCLC.

## Introduction

Lung cancer is the leading cause of cancer-related mortality in men and the second among women worldwide.[1] With annually about 1.3 million new registered non-small cell lung cancer (NSCLC) cases, every effort should be made towards finding more personalized cancer therapies[2].

**Fig 1:**
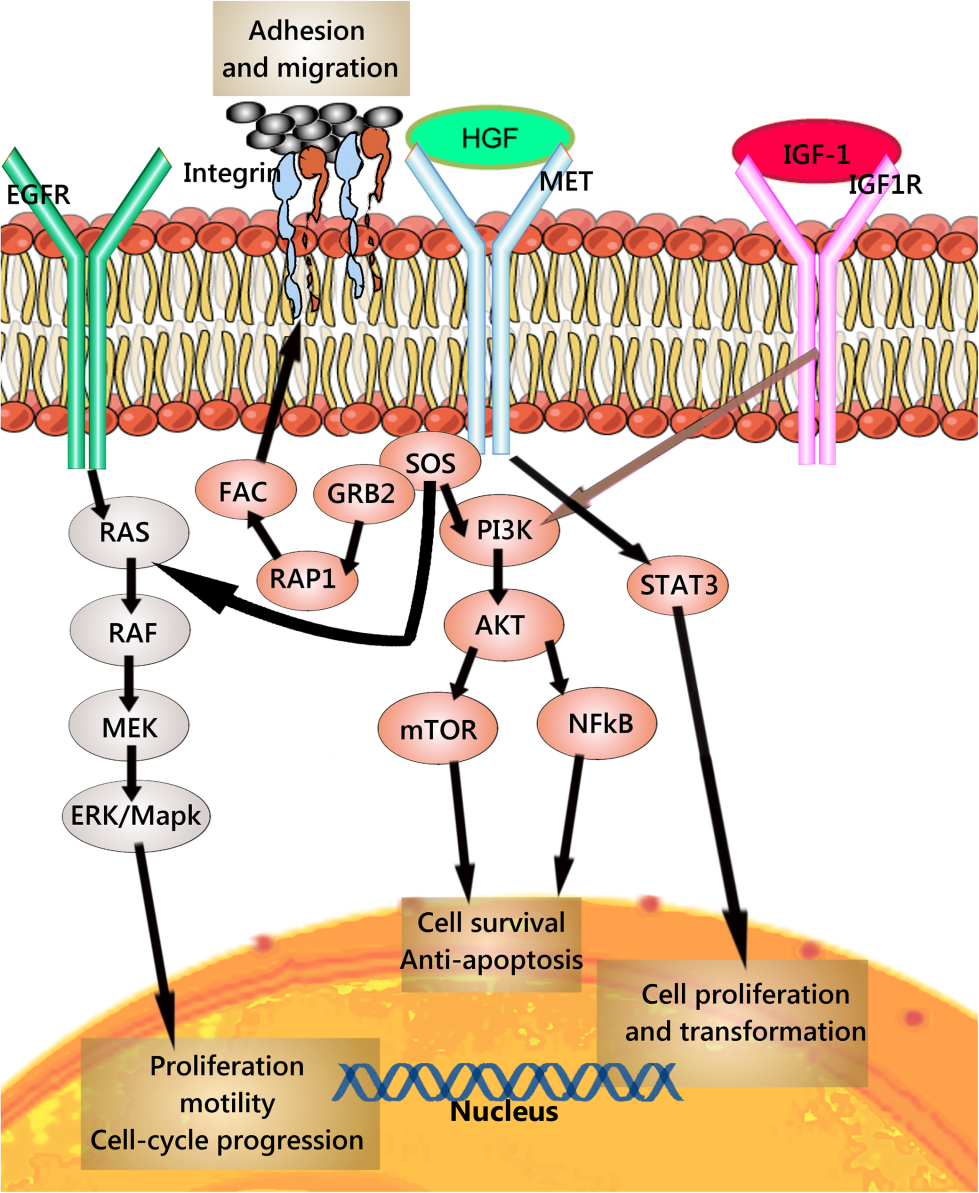
Simplified schema of the MET signaling adaptors and mediators. Activation of the MET receptor by its ligand triggers tyrosines within the multifunctional docking site to become phosphorylated and recruit signaling effectors, including the adaptor protein growth factor receptor-bound protein 2 (GRB2). The MET pathway is modulated by cell surface molecules, including the HER family (HER1, HER2 and HER3) and IGF1R.MET can activate the effector molecule phosphatidylinositol 3-kinase (PI3K), and signals through the AKT/protein kinase B axis, which activates the mammalian target of rapamycin (mTOR) axis stimulating cell growth and protein synthesis. Moreover, the activation of either PI3K-AKT can activate NF-κB, which then can be released and stimulate gene transcription after being translocated to the nucleus.MET activation can result in the down-stream activation of the RAS-MAPK pathway. The nucleotide exchanger protein Son of Sevenless (SOS) activates the rat sarcoma viral oncogene homolog RAS by binding with the GRB2 (GRB2-SOS complex). This complex can activate the v-raf murine sarcoma viral oncogene homolog B1 (RAF) kinases, which successively activate MAPK effector kinase (MEK), and finally results in the activation of the mitogen-activated protein kinase (MAPK).Activation of MET cytoplasmic signalling cascades can additionally alter cell invasiveness, motility, and cytoskeleton, mediated through the RAS-related protein 1 (RAP1), the focal adhesion complex (FAC) as well as integrin connections. MET can additionally activate the downstream axis of the Signal transducer and activator of transcription 3 (STAT3) pathway. STAT3 can be activated through phosphorylation after binding directly to MET resulting in dimerization and translocation to the nucleus and consequently mediating cell proliferation, transformation, as well as tumorigenesis and invasion. [6–9].

MET (the hepatocyte growth factor receptor, HGFR, also known as c-Met, AUTS9; RCCP2; DFNB97, and as mesenchymal-epithelial transition factor) is activated by its ligand HGF and exerts broad biological effects associated with malignancy including cell proliferation, cell scattering and migration, induction of cell polarity, and angiogenesis.[3] MET is reported to regulate the morphogenesis of both epithelial and stromal cells [4], in addition to its role in the mesenchymal-epithelial transition of cells, and to play an essential role in tissue repair[5] (Fig 1).

MET has recently also gained ground as an important target in the treatment of malignancy. Using the MET inhibitor onartuzumab plus erlotinib, an improved progression-free survival (PFS) and a better overall survival (OS) was seen in immunohistochemically MET-positive NSCLC patients, while a worse outcome was observed in MET-negative patients treated with onartuzumab.[10] However, recent published results by Spigel et al [11]from the III randomized trial of Onartuzumab plus Erlotinib versus Erlotinib trial failed to show similar results. Other trials are still ongoing[6, 12].

Herein, we conducted a study investigating the prevalence and the prognostic role of MET in NSCLC. We compared the expression of MET as detected by immunohistochemistry (IHC) with its gene amplification by means of silver *in situ* hybridization (SISH). Due to observations reporting MET activation and treatment resistance following anti EGFR treatment,[12, 13] we aimed to immunohistochemically study the prevalence and possible correlation between MET expression and two major forms of mutant *EGFR*; E746-A750deletion mutation of exon 19 (*EGFR*del) and the single L858R deletion mutation of exon 21 (*EGFR*mut). In the light of studies indicating IGF1R as a putative coactivator of MET (Fig 1), we investigated the prevalence and the prognostic significance of *IGF1R* gene using SISH and the ligand IGF-1 using immunohistochemistry. Finally, we investigated the correlation between polysomy of chromosome 7, on which the *MET* gene is located, and disease-specific survival (DSS) in patients with NSCLC.

## Materials and methods

### Patients and clinical material

This retrospective study utilized primary tumor tissue from patients diagnosed with NSCLC stage I–IIIA; the tissue was surgically resected at the University Hospital of Northern Norway and Nordland Central Hospital between 1990 and 2004. Three hundred seventy-one patients were registered from the hospitals’ databases. The following exclusion criteria were employed: (1) radiotherapy or chemotherapy prior to surgery, (2) other malignancy within 5 years before the NSCLC diagnosis and (3) inadequate paraffin-embedded tissue blocks. Thirty-six patients fell into these three categories (criteria 1: n = 10; criteria 2: n = 13; criteria 3: n = 13) and were excluded from the study. Adjuvant chemotherapy had not yet been introduced as a therapeutic option in Norway during this time span (1990–2004). In total, 335 patients with complete medical records and adequate paraffin embedded tissue blocks were included in this study. The tumors were subtyped and histologically graded according to the recent World Health Organization (WHO) guidelines.[14] The patients were staged corresponding to the 7th edition of the UICC TNM classification, where 9 patients were regarded as having an *in-situ* disease regarding the new lung cancer classification resulting in a total of 326 patients eligible for this study.[15] The Regional Committee for Medical and Health Research Ethics, as well as the Norwegian Data Inspectorate, approved this study.

### Microarray constructions

Two experienced pathologists (S.A.S. and K.A.S.) investigated all the lung cancer specimens thoroughly. Tissue microarray (TMA) blocks were constructed using a tissue-array instrument (Beecher Instruments, Silver Springs, MD, USA) as previously described [16].

### Immunohistochemistry and silver *in situ* hybridization

The applied antibodies have been previously subjected to in-house validation by the manufacturer for IHC analysis of paraffin-embedded material. The antibodies used in this study were as follows: Phospho-MET Receptor (1:160; rabbit monoclonal, clone D26; #3077; Cell Signaling Technology,Danvers, MA, USA). EGF Receptor (E746-A750del Specific; 1:100; rabbit monoclonal, clone D6B6; #2085; Cell Signaling Technology, Danvers, MA, USA). EGF Receptor(L858R Mutant Specific; 1:100; rabbit monoclonal, clone 43B2; #3197; Cell Signaling Technology, Danvers, MA, USA).*IGF1R* gene and Chromosome 15 probe (prediluted by the manufacturer; INFORM IGF1R DNP Probe: 800–4458 and INFORM Chromosome 15 DIG Probe: 800–4459; Ventana Medical Systems, Illkirch, France). *MET* gene and chromosome 7 probe (prediluted by the manufacturer; INFORM MET DNA Probe: 800–4372; 05575311001 and INFORM Chromosome 7 Probe: 800–4342; 05278899001). IGF-I (1:100, rabbit polyclonal, clone H-70; #sc-9013; Santa Cruz Biotechnology incorporated, 10410 Finnell Street, Dallas, Texas 75220, USA). The detailed methodology for immunohistochemistry and silver *in situ* hybridization has been previously published[16, 17].

### Scoring of immunohistochemistry (IHC) and silver *in situ* hybridization (SISH)

**Fig 2:**
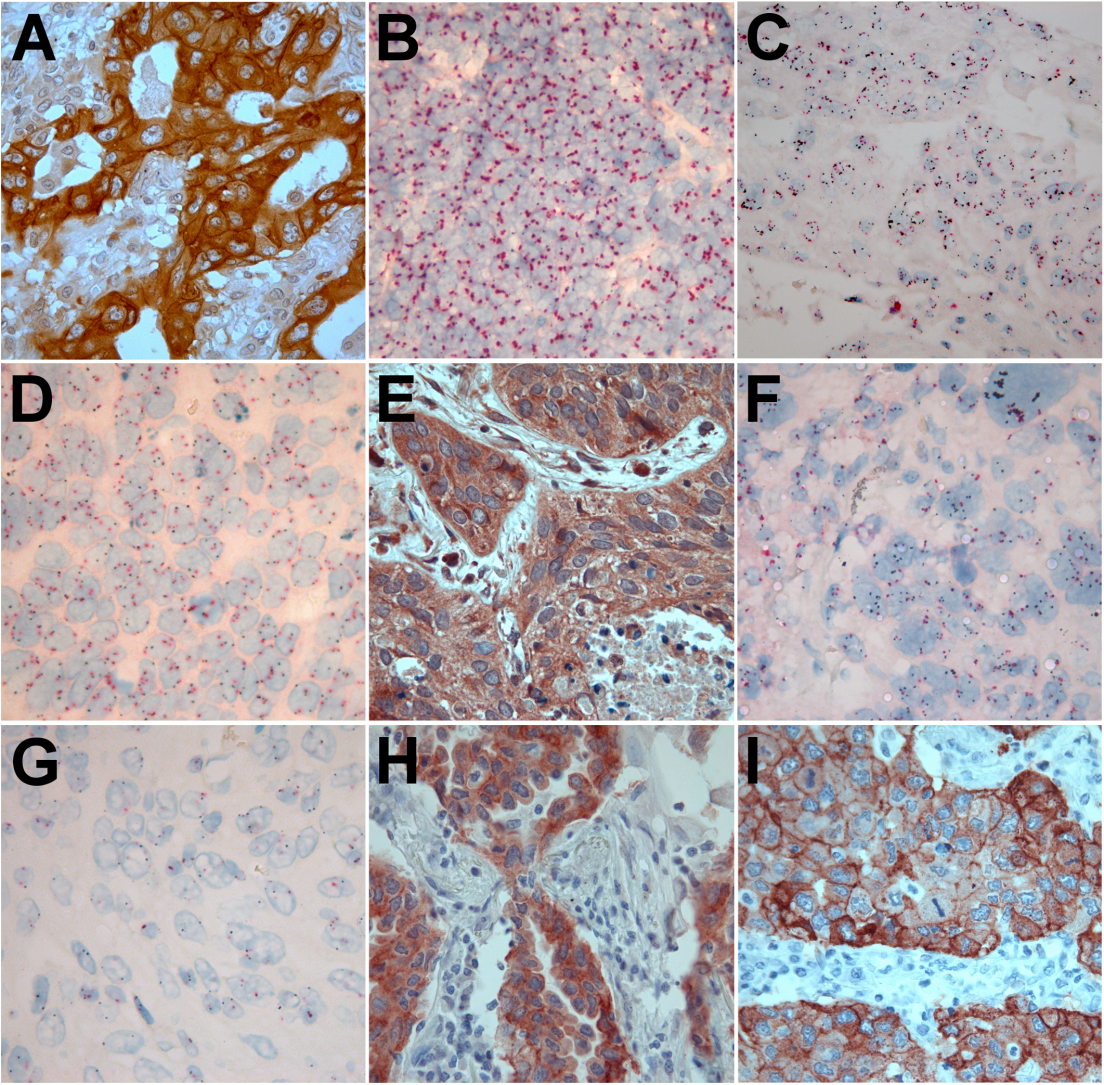
Expression of investigated markers in NSCLC tissues. A) High expression of MET in a patient suffering an adenocarcinoma. B) Tumor tissue with a high chromosome 7 count on which the *MET gene* is located. C) Tumor tissue with high *MET* gene copy number count. D) Balanced *MET* gene copy number to chromosome 7 count ratio. E) High IGF-1 IHC expression of a male patient with squamous cell carcinoma. F) Tumor tissue in a male patient with a high *IGF1R* gene copy number to chromosome 15 count. G) Tumor tissue in a male patient with a balanced *IGF1R* gene copy number to chromosome 15 count. H) An adenocarcinoma with positive IHC staining for the *EGFR* gene mutation E746-A750 deletion of exon 19. I) An adenocarcinoma with positive IHC staining for the *EGFR* gene single L858R deletion mutation of exon 21.

The tissue cores were scored by light microscopy to determine the degree of cytoplasmic and nuclear expression. Examples of various markers’ expressions are shown (Fig 2). Staining for *MET* and *IGF1R* genes resulted in signals as black dots on the corresponding chromosomes for both genes, while centromeres of chromosome 7 and 15 were stained as red dots. Regarding SISH scoring, uniform guidelines exist and were strictly followed for the interpretation of gene and chromosome signals.[18]Even though these guidelines were developed for breast cancer testing, we found similar staining results in NSCLC biopsies. Evaluation of*HER2*sish is reported in breast cancer as the ratio of the average number of *HER2* gene copies to the average number of chromosome 17 copies (HER2:chr17) per cell. Because no clear guidelines have been established for measuring *MET or IGF1R*gene amplification in NSCLC, we also sought to determine whether the absolute number of *MET and IGF1R*gene copies detected by SISH (i.e., the number of black dots observed in the nuclei of tumor cells) would add prognostic significance beyond that established by the gene copy number to chromosome count ratio. From each tumor, four cores were eligible for scoring. In each core, we counted genes and centromere signals in 20 cells at least in two cores, where one core was taken from the central part of the tumor and the other core was taken at the advancing edge of tumor. An overall average was taken for both gene and centromere count. The other two cores included for the most stromal tissue surrounding epithelial cells of NSCLC. Heterogeneity was not observed while scoring immunostains. Regarding SISH, in cases of heterogeneity hot spots with the highest gene or centromere count were scored. The number of gene copies was assessed according to the manufacturer’s protocols for INFORMHER2 DNA. Briefly, a discrete dot was counted as a single copy of *MET*, *IGF1R*, chromosome 7 or chromosome 15. Some nuclei showed multiple discrete copies. Clusters of dots representing many copies of the targetgenes were also apparent; a small cluster of multiple signals was counted as six copies and a large cluster was counted as 12 copies.

Staining for MET, IGF-1, EGFRmut and EGFRdel resulted in a homogenous cytoplasmic staining (Fig 2). Due to homogenous staining, there was no need to score the density of positive cells. Scoring the intensity of staining was considered as representative to measure the protein expression by immunohistochemical staining. Tissue sections for the two first mentioned proteins were scored semi quantitatively for staining intensity in tumor epithelial cells as follows: 0 = negative, 1 = weak, 2 = intermediate, and 3 = strong. Regarding the immunohistochemical expression for IGF-1 and MET, tumors showing intermediate or strong positivity were regarded as the high expression group, while tumors with weak or negative staining were regarded as the low expression group. When evaluating EGFRmut and EGFRdel, only clear strong positive tumors where regarded as harboring gene mutations.

All of the anonymized samples were semi quantitatively and independently scored by two experienced pathologists (S.A.S. and E.R). In the event of disagreement, the slides were re-examined and a consensus was reached by the observers. When assessing one variable for a given core, the observers were blinded to the scores of the other variables and the outcome. The reproducibility of the IHC and SISH evaluation in randomly selected specimens is high. The IHC and SISH scores from each observer were compared for interobserver reliability using a 2-way random effects model with absolute agreement definition, yielding an intra-class correlation coefficient (reliability coefficient) and Cohen’s kappa. There was an excellent scoring agreement for two tested markers (*MET* SISH and EGFRdel) between the pathologists, with an intra-class correlation coefficient of 0.91 (P < 0.001) for *MET* SISH and 0.93 (P < 0.001) for EGFRdel.

### Statistical analyses

The statistical analyses presented in this study were performed using the statistical package IBM SPSS, version 24 (SPSS Inc., Chicago, IL, USA).Chi-square and Fisher’s exact test were used to examine the correlation among different molecular markers and clinicopathological factors. The *r*-values represent Spearman’s rank correlation coefficients. The Kaplan–Meier method was used for drawing curves for univariate analysis of the association between marker expression and disease-specific survival (DSS). DSS was determined from the date of surgery until the time of lung cancer death. Statistical significance between the survival curves was assessed utilizing the log-rank test. Cut-offs were chosen by a minimal p-value approach, with regard to the association between markers and survival endpoints. The survival curves were terminated at 120 months as fewer than 10% of patients were at risk after this point. Statistically significant variables from the univariate analysis were included in the multivariate analysis, applying the Cox proportional hazards model. The data were run in a backward stepwise Cox regression with a probability for stepwise entry and a removal set at 0.05 and 0.10. The significance level was set at a P-value less than 0.05.

## Results

### Clinicopathological variables

We retrospectively examined a non-selected group of NSCLC patients, which was examined in previous works by our research group.[17]Clinical, demographic and histopathological variables are presented in Table 1.Of the 326 NSCLC patients, the majority were male (76%) and nearly all (96%) were previous or present smokers. The median age was 67 years (range 28–85 years) and the median follow-up of survivors was 105 months (range 73–234 months). Histologically subtypes presented as: (58.6%) 191 cases of squamous cell carcinoma (SCCs), (31.9%) 104 cases of adenocarcinomas (ACs) and (9.5%) 31 cases of large cell (anaplastic) carcinomas (LCCs).Fifty-nine patients (18%) were administered adjuvant radiotherapy due to nodal metastasis or non-radical surgical margins verified during surgery. The median follow-up of the survivors was 105 months (range 73–234 months).

**Table 1:** Prognostic clinicopathologic variables as predictors for disease-specific survival in 326 NSCLC patients (univariate analyses; log-rank test).

Characteristic	Patients (n)	Patients (%)	Median survival (months)	5-Year survival (%)	P
**Age**					
≤ 65 years	151	46	98	65	0.44
> 65 years	175	54	NR	78	
**Sex**					
Female	78	24	190	63	0.20
Male	248	76	83	56	
**Smoking**					
Never	13	4	19	41	0.28
Current	210	64	NR	60	
Former	103	32	84	54	
**Performance status**					
ECOG 0	189	58	NR	62	**0.029**
ECOG 1	119	36	69	53	
ECOG 2	18	6	25	33	
**Weight loss**					
< 10%	294	90	127	58	0.79
> 10%	32	10	98	57	
**Histology**					
SCC	191	59	NR	66	**0.013**
AC	104	32	52	45	
LCC	31	9	98	56	
**Differentiation**					
Low	138	42	47	47	**< 0.001**
Moderate	144	44	190	66	
Well	44	14	NR	65	
**Surgical procedure**					
Lobectomy + Wedge*	238	73	190	61	**0.004**
Pneumonectomy	88	27	37	47	
**Pathological stage**					
I	203	62	190	69	**< 0.001**
II	91	28	41	43	
IIIa	32	10	18	19	
**Tumor status**					
1	84	26	190	75	**0.002**
2	215	66	74	53	
3	27	8	47	35	
**Nodal status**					
0	223	69	190	66	**< 0.001**
1	76	23	35	43	
2	27	8	18	18	
**Surgical margins**					
Free	300	92	190	58	0.29
Not free	26	8	47	47	
**Vascular infiltration**					
No	275	84	190	58	**< 0.001**
Yes	51	16	27	32	

NR, not reached

*Wedge, n = 10

Abbreviations: SCC, squamous cell carcinoma; AC, adenocarcinoma; LCC, large-cell carcinoma

### Biomarker expression and correlation in NSCLC tissue

Of 326 cases, all were immunohistochemically evaluable for the expression of MET and IGF-1, while 295 were evaluable for the silver *in situ* hybridization analysis of the *MET* gene copy number on chromosome 7, and 237 cases were eligible for the *IGF1R* gene copy number analysis on chromosome 15.

For the immunohistochemical analysis of *EGFR*gene mutations, 313 cases were eligible for EGFRmut, while 318 cases were eligible for the detection of the EGFRdel mutation.

MET, IGF-1, EGFRmut and EGFRdel showed a homogenous cytoplasmic staining pattern.

We found high (moderate to strong) cytoplasmic expression of MET and IGF-1 in 82.8% and 6.1% of valid tumor samples, respectively. The reported prevalence of phosphorylated MET in our material is consistent with results demonstrated by other investigators. [19–21]. However, a lower grade of prevalence has been observed by other reports [22–24]. Using various methods to detect the prevalence of phosphorylated MET in FFPE tissue, Dua et al[25] demonstrated that their c-MET FFPE assay could detect and quantify c-MET receptor levels in FFPE tumor specimens, and that these measurements would correlate well with measurements obtained by conventional methods.

A high chromosome 7 count, higher than 2 copies was found in 21.5% of valid tumor samples, while 6% of tumor samples showed polysomy, i.e. a chromosome 7 count higher than 3. A *MET* gene to chromosome 7 ratio higher than 1 was observed in 7.1% of valid tumors, while an *IGF1R* gene to chromosome 15 ratio higher than 1 was observed in 21.6% of valid tumors. In the male cohort population, 6.1% of valid tumors showed an *IGF1R* gene to chromosome 15 ratio higher than 1, p = 0.015, while this was observed in 15.5% of all females with NSCLC, p = 0.021. An EGFRdel mutated protein indicating a gene mutation was found in 6% of all valid patients (5.8% of males and 6.6% of women) while 5.4% showed an EGFRmut mutated protein, indicating a mutated gene (4.6% of males and 8% of women). Prevalence of investigated factors is shown (Table 2).The above-mentioned markers did not correlate with age, gender, smoking, WHO performance status, or vascular infiltration.

**Table 2:** Prognostic Effect of MET, *MET* gene copy number, polysomy of chromosome 7, *IGF1R* gene copy number and IGF-1 Expression in Tumor Epithelial Cells of primary NSCLC in 326 patients (univariate analysis; log-rank test).

Marker expression	Patients (n)	Patients (%)	Median survival (months)	5-Year survival (%)	*P*
**MET cytoplasmic in adenocarcinoma**					**0.026**
Low	19	18	NR	68	
High	85	82	47	58	
***MET* gene /chromosome 7 ratio**					**0.005**
Low	21	7	35	36	
High	274	93	190	59	
**Polysomy of chromosome 7 (>3 copies)**					**0.002**
Low	280	94	190	60	
High	18	6	37	11	
***IGF1R* gene/chromosome 15 ratio**					**0.037**
Low	217	92	190	61	
High	20	8	37	40	
**IGF-1 cytoplasmic**					0.053
Low	270	83	138	55	
High	56	17	179	65	
**EGFR mutation (E764-A750del)**					0.498
Present	19	6	127	58	
Absent	299	94	NR	62	
**EGFR mutation (L858R)**					0.628
Present	17	5	NR	60	
Absent	269	95	127	58	

### Univariate analysis

Results from the univariate analysis regarding the clinical variables and their impact on DSS are presented in Table 2. T-stage (P<0.001), N-stage (P<0.001), pathological stage (P<0.001), WHO performance status (p = 0.016), histology (P = 0.028), vascular infiltration (P = 0.001), differentiation (p<0.001) and surgical procedure (p = 0.007) were significant prognosticators for the total patient population.

**Fig 3:**
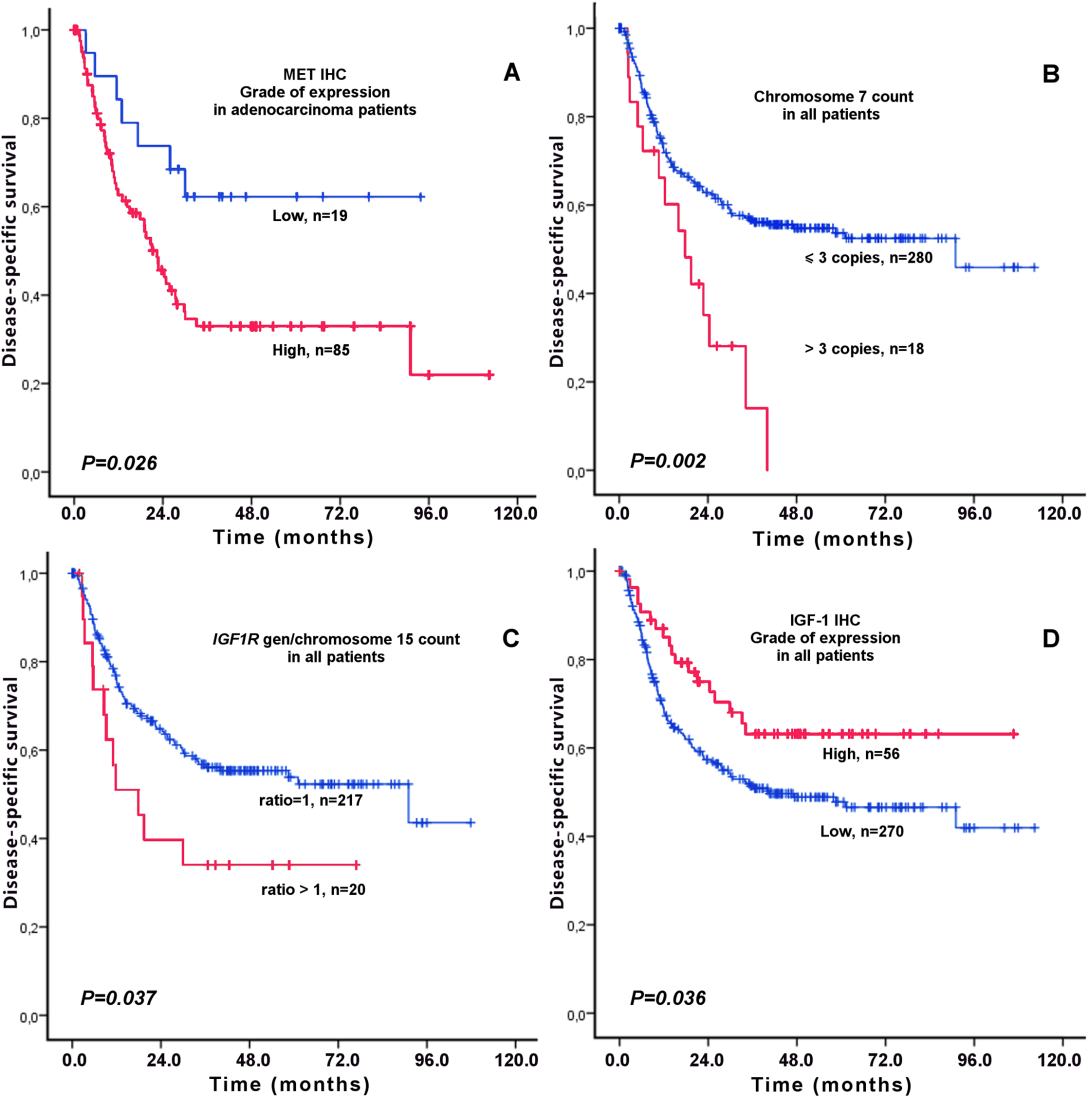
Kaplan–Meier curves of disease-specific survival. Survival curves according to (A) MET immunohistochemical expression in patients with adenocarcinoma (B) Chromosome 7 copy number count in patients with NSCLC (C) *IGF1R* gene copy number to chromosome 15 ratio in NSCLC patients, and (D) IGF-1 immunohistochemical expression in patients with NSCLC.

High cytoplasmic MET expression showed a significant negative prognostic effect only in patients with adenocarcinoma (p = 0.026), but not for the whole cohort (p = 0.411; Table 3 and Fig 3).*MET* gen copy count to chromosome 7 ratio>1 was a significant positive prognostic marker (p = 0.005). We sought to determine if different cut-off points for the *MET* gene/chromosome 7 ratio would provide an additional prognostic significance. A *MET* gene/chromosome 7 ratio >1.5 (p = 0.21) did not show a prognostic significance. The same applies for a *MET* gene/chromosome 7 ratio >2 (p = 0.43). However, the polysomy of chromosome 7 emerged as a highly specific (p = 0.002) negative prognosticator for all patients.*IGF1R*gene copy number was a highly negative prognostic marker for all NSCLC patients (p = 0.037), but was even more significant in males (p = 0.015) than in females (p = 0.021).Finally, a high IGF-1 expression showed a trend as a negative prognostic marker in males (p = 0.053) but not for the whole cohort population. There was no significant correlation between DSS and tumor epithelial cell expression of the *EGFR* gene mutations EGFRmut (p = 0.628) and EGFRdel (p = 0.498).

**Table 3:** Results of Cox regression analysis summarizing significant independent prognostic factors for disease-specific survival.

Factor in all NSCLC patients	Hazard Ratio	95% CI	P
**Chromosome 7 (copy number)**			0.011*
Up to 3 chromosome copies	1.00		
Higher than 3 chromosome copies	2.29	1.21–4.35	**0.01**
**Tumor differentiation**			0.001*
Well	1.00		
Moderate	1.77	0.83–3.79	0.14
Poor	0.73	0.33–1.65	0.45
**Performance status**			0.001*
ECOG0	1.00		
ECOG1	1.87	1.19–2.93	**0.007**
ECOG2	3.54	1.62–7.73	**0.002**
**Pathological stage**			0.0001*
I	1.00		
II	1.77	1.1–2.85	**0.019**
IIIA	3.69	1.94–7.03	**0.0001**
**Factor in patients with adenocarcinoma**	**Hazard Ratio**	**95% CI**	**P**
**MET cytoplasmic**			0.013*
Low	1.00		
High	2.85	1.25–6.50	**0.013**
**Performance status**			0.0001*
ECOG0	1.00		
ECOG1	2.80	1.58–4.96	**0.0001**
ECOG2	16.22	4.32–60.94	**0.0001**
**Pathological stage**			0.0001*
I	1.00		
II	5.42	2.90–10.15	**0.0001**
IIIA	2.13	0.09–5.09	**0.09**
**Vascular infiltration**			0.015*
Absent	1.00		
Present	2.65	1.21–5.81	**0.015**
**Factor in males with NSCLC**	**Hazard Ratio**	**95% CI**	**P**
***IGF1R* gene/chromosome 15 ratio**			0.018*
1	1.00		
>1	2.67	1.18–6.00	**0.018**
**Tumor differentiation**			0.005*
Well	1.00		
Moderate	1.41	0.58–3.41	0.451
Poor	0.57	0.22–1.49	0.247
**Performance status**			0.018*
ECOG0	1.00		
ECOG1	2.07	1.24–3.46	**0.005**
ECOG2	1.72	0.70–4.20	0.236
**Pathological stage**			0.0001*
I	1.00		
II	1.53	0.90–2.58	0.083
IIIA	4.93	2.24–10.84	0.114
**Vascular infiltration**			0.0001*
Absent	1.00		
Present	3.55	1.9–6.61	**0.0001**

* Overall significance as a prognostic factor

### Multivariate Cox proportional hazards analysis

Significant clinic pathological and molecular variables from the univariate analyses were entered into the multivariate analysis. The statistically significant results are presented in Table 3.

Polysomy of chromosome 7 in tumor cells correlated significantly and independently with a poor prognosis (HR: 2.29; 95% CI: 1.21–4.35; p = 0.011). In patients with adenocarcinoma, a high cytoplasmic MET expression was an independent negative prognostic factor (HR: 2.85; 95% CI: 1.25–6.50; p = 0.013). In males with NSCLC a high *IGF1R* gene copy number to chromosome 15 count ratio higher than one, was significantly and independently correlated to a poor prognosis (HR: 2.67; 95% CI: 1.18–6.00; p = 0.018). For the whole cohort, the histologic subtypes did not reach a statistically prognostic significance, neither for the group of adenocarcinoma (p = 0.165), nor for the group of squamous cell carcinoma (p = 0.7) or for the large cell carcinoma group (p = 0.466).

## Discussion

The chief aim of our study was to investigate the prognostic role of MET expression and the *MET* gene copy number gain in NSCLC. In addition to IHC we used SISH to investigate the *MET*gene. While, in the adenocarcinoma patient population, independent of other clinicopathological variables, a high cytoplasmic MET expression was a significant negative prognosticator, as determined by IHC, a similar correlation was not found in patients with *MET* gene copy number gain, i.e. the absolute *MET* gene copy number in tumor cells did not affect the prognosis. Surprisingly, investigating the ratio of *MET* gene to chromosome 7, we found a trend of better survival in patients with a higher ratio in the whole cohort in the univariate analysis, but not in the multivariate analysis. However, a high *MET* gene to chromosome 7 ratio appeared to be a rather non-frequent event. We observed a *MET* gene to chromosome 7 ratio higher than 1 in 7.1% of our non-selected patients. This is in agreement with recent published data by Noonan et al[26], where–using fluorescence in situ hybridization in lung adenocarcinoma- they observed a *MET* gene to chromosome 7 ratio of 1.8 or higher only in 4.5% of adenocarcinoma patients. The rather confusing positive prognostic effect of a high *MET* gene to chromosome 7 ratio led us to investigate a possible role of chromosome 7 polysomy in NSCLC patients. Interestingly, we found a high chromosome 7 count, higher than three chromosome copies as a highly independent negative prognostic factor for the whole cohort population. We assumed that a dysfunction in the transcriptional or posttranscriptional controlling mechanisms could partly explain the discrepancies between the IHC and SISH results; still we wanted to explore other genes located on chromosome 7. Chromosome 7 is known to harbor genes whose alteration my play an important role in multiple diseases as cystic fibrosis[27], but also in tumorigenesis [28]with over than 1150 protein- coding genes, 605 of which have been validated by transcript sequences.[29] Nevertheless, three genes; *EGFR*, *MET*, and *BRAF* emerge as of special interest in NSCLC. Both *MET* and *EGFR* gene amplification are described to have a critical predictive role in NSCLC. [30]*BRAF* appears to have a role as a predictive marker in patients with advanced melanoma disease [31, 32], with a rather limited therapeutic effect followed by resistance development in NSCLC patients.[33] There are reports [34–36] proposing a mechanism for MET and EGFR axis regulation mediated by miRNAs. Additionally, MET protein activation has been associated with primary resistance to EGFR tyrosine kinase inhibitor (TKI) therapy in NSCLC patients (Fig 1).[13, 37] These observations have initiated a scientific debate about novel bispecific EGFR/MET inhibitors to obtain better therapeutic results. [10, 38, 39]In the light of this, we sought to determine any correlation between MET expression, *MET* gene copy number count (GCNC) and two of the most observed *EGFR* gene mutations in NSCLC; *EGFR*mut and *EGFR*del [40, 41]in untreated patients. A critical issue in investigating EGFR mutations using immunohistochemistry was finding antibodies with an acceptable sensitivity and specificity. The specificity of the used antibodies has been certified by the manufacturer both by means of Western blot and flow cytometric analysis[42, 43] Additionally, Several groups[44–46] have concluded with an acceptable specificity and sensitivity of the aforementioned antibodies. Brevet et al. [47] reported that IHC using the *EGFR* L858R specific antibody showed a sensitivity of 95.2% and a specificity of 98.8%. They further found that the *EGFR* exon 19 mutant specific antibody would detect 100% of 15-bp (base pair) deletions with a high specificity, however, a significant lower sensitivity of about 48.6% in non-15-bp exon 19 deletions was observed. According to the COSMIC database, Non 15-bp exon 19 deletions account for about 35% of exon 19 deletions[48].

We did not find a statistic correlation between MET expression, *MET* GCNC and any of the examined *EGFR* gene mutations. Further, none of the investigated *EGFR* mutations had a prognostic significance, despite conflicting results by other groups[40, 49, 50].

The essential role of the HGF-MET cellular pathway has been further established following observations on *MET*-null mutant mice embryos, with malformation of liver, placenta,[7] melanocytes,[51] and testis.[52] Meanwhile, it has been observed that overexpression of MET can have an oncogenic potential by itself and can induce hepatocellular carcinoma in liver cells [53]. There is established evidence [54] that MET is sufficient for transformation of normal human osteoblasts causing an osteosarcoma-like disease *in vivo*. Furthermore, dysregulation of the HGF-MET pathway has been demonstrated in malignancies of epithelial cell origin, represented by carcinomas of the lung, mamma, hepatic cells, pancreas ovaries, papillary renal carcinoma, papillary thyroid carcinoma, and carcinomas of the colorectal system.[6]Dysregulation of the HGF-MET cellular axis may due to *MET* gene mutations, *MET* amplification, chromosomal rearrangement, MET transcriptional upregulation or changes in the autocrine or paracrine signaling. Several studies have investigated the prognostic role of the MET receptor and the *MET* gene alteration in NSCLC. While some groups investigated a rather small group of patients [55–57], other groups found a negative prognostic effect of high MET protein expression and *MET* gene copy number gain either independent of the histologic type[58–63] or only in patients with adenocarcinoma[64] or squamous cell carcinoma[65].

Exploring both the MET protein expression and the *MET* GCNC in 140 NSCLC patients, Dziadziuszko et al[66] concluded that neither was associated with prognosis. Meanwhile, Tran et al[67] observed that MET overexpression and *MET* high (GCNC) occur in a low proportion of primary NSCLCs and are associated with a good prognosis. Awad et al [68]reported MET exon 14 mutations to occur in rather older patients and that they may represent a clinically unique molecular subtype and a possible important therapeutical target in NSCLC. Preliminary findings from the PROFILE 1001 trial [69] show that crizotinib demonstrates a meaningful antitumor activity in patients with NSCLC harboring *MET* exon 14 alterations. Similar results were also recently published by Lu et al[70].

Recent studies have proposed a ligand-independent MET activation.[71, 72] Using prostate cancer cell lines, Varkaris et al,[73] proposed a full but rather delayed activation of MET through IGF1R (Fig 1). Consequently, we aimed to determine a possible correlation between the MET receptor expression, *MET* GCNC, and the IGF-1 axis.

MET receptor expression did not show a significant correlation with the *MET* GCNC (p = 0,77), the IGF1R GCNC (p = 0.64) or IGF-1 expression (p = 0.31).

However, investigating the prognostic significance of IGF-1 expression in epithelial NSCLC showed a trend towards worse survival. Furthermore, investigating the ratio of *IGF1R* GCNC to chromosome 15, this ratio immerged as a highly significant and independent negative prognostic indicator for disease specific survival in males, whereas it did not show prognostic influence in females. Gender differences in cancer tumorigenesis and survival are most likely associated with different sex hormone effects on various genes. While there are promising preliminary results among breast cancer and NSCLC patients treated with the IGF1R inhibitor Dalotuzumab,[74] according to our results, the subgroup of male patients with NSCLC appears to benefit the most of such a treatment. Promising preclinical trials investigating the role of IGF1R as a therapeutical target has resulted in the initiating of clinical trials on patients with multiple myeloma.[75]There are studies[76] suggesting IGF1R as a potential target in NSCLC treatment. While Tsuata et al[77] and Capuzzo et al[78]reported that the IGF1R expression did not represent a prognostic factor in resected NSCLC patients, other research groups[79–81] have either found a negative prognostic significance of high IGF1R expression or *IGF1R* GCNC.[82] However, future results of stratified treatment-trials among NSCLC patients will be needed to fortify these results.

Our current prognostic findings as detected by SISH for *IGF1R* and by immunohistochemistry for MET have a relevant practical implementation. While *IGF1R* SISH analyses seems to give additional information about the subgroup of NSCLC patients, who most likely would benefit of an anti-IGF1R therapy regimen, the *MET* SISH analyses seem to be biased when investigating the ratio of gene copy number to chromosome count due to the highly negative prognostic significance of chromosome 7 polysomy, higher than 3 chromosome copies. Our results would most probably apply to any SISH analysis investigating a gene to chromosome ratio located on chromosome 7. Even thought our findings would apply to small subgroups of patients, still finding therapeutic aid to these subgroups would be regarded as a significant step towards personalized NSCLC treatment. Until this, every effort should be made to find more specific and even more personalized potential molecular targets whose status in tumor samples might impact therapeutic responses.

## Supporting information

S1 Table Minimal data set. Data set with scoring results for all the markers included in the study.
(SAV)
